# An Appreciation of Anatomy in the Molecular World

**DOI:** 10.3390/jcdd7040044

**Published:** 2020-10-15

**Authors:** Bjarke Jensen, Vincent M. Christoffels, Antoon F. M. Moorman

**Affiliations:** Department of Medical Biology, Amsterdam Cardiovascular Sciences, University of Amsterdam, Amsterdam UMC, Meibergdreef 15, 1105AZ Amsterdam, The Netherlands; v.m.christoffels@amsterdamumc.nl (V.M.C.); a.f.moorman@amsterdamumc.nl (A.F.M.M.)

**Keywords:** heart development, cardiac conduction system, cardiac structure

## Abstract

Robert H. Anderson is one of the most important and accomplished cardiac anatomists of the last decades, having made major contributions to our understanding of the anatomy of normal hearts and the pathologies of acquired and congenital heart diseases. While cardiac anatomy as a research discipline has become largely subservient to molecular biology, anatomists like Professor Anderson demonstrate anatomy has much to offer. Here, we provide cases of early anatomical insights on the heart that were rediscovered, and expanded on, by molecular techniques: migration of neural crest cells to the heart was deduced from histological observations (1908) and independently shown again with experimental interventions; pharyngeal mesoderm is added to the embryonic heart (1973) in what is now defined as the molecularly distinguishable second heart field; chambers develop from the heart tube as regional pouches in what is now considered the ballooning model by the molecular identification of regional differentiation and proliferation. The anatomical discovery of the conduction system by Purkinje, His, Tawara, Keith, and Flack is a special case because the main findings were never neglected in later molecular studies. Professor Anderson has successfully demonstrated that sound knowledge of anatomy is indispensable for proper understanding of cardiac development.

## 1. Introduction

Single cell sequencing of whole hearts of adults and embryos reveals a stunning complexity [1,2] that could not have been anticipated on the basis of the dominant disciplines of heart research of the 20th century, anatomy and electrophysiology. History shows that over time some techniques become obsolete and some disciplines, such as morphology, disappear [3]. Not everything, however, gets supplanted in the brave new world. Instead, problems in biology and medicine are addressed by an increasing diversity of tools and approaches as revealed by the interdisciplinarity of recent studies [4,5,6]. Concerning cardiac pathologies, the criteria for structural assessments of hypertrophy, wall thinning, excessive trabeculae, and much more have not changed much while imaging has undergone profound developments [7]. This testifies to the persistent role of anatomy in biomedical investigations of the heart.

Here, we provide examples of highly perceptive discoveries made within anatomy that were later rediscovered, and much elaborated on, using technological approaches developed in the fields of developmental and molecular biology. As we go back in time, the descriptions become fewer and less detailed and at some point, one can reasonably ask whether the original descriptions in fact concern the same matter as described in current research. Our primary aim is not to establish who made the original descriptions. Rather, we want to highlight a few cases to emphasize the importance of complementary approaches. The reason for the emphasis on anatomy is to celebrate our dear friend Bob Anderson.

## 2. Early and Highly Perceptive Insights

### 2.1. Neural Crest Cells

**Figure 1:**
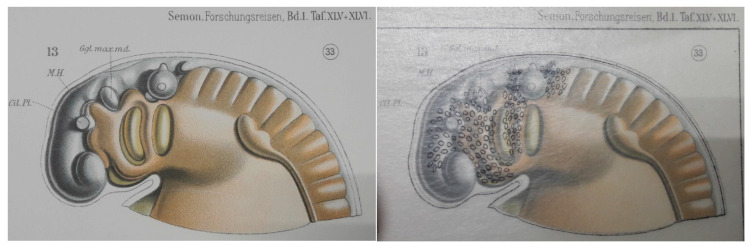
Neural crest cells migrating down the pharyngeal arches of a stage 33 embryonic Australian lungfish. Transparent overlay paper was used in this description from 1908 to show the migration (black ovals in right-hand image represent the positions of neural crest cells). Photographed from [8].

In 1908 the first of two large volumes appeared on the development of the Australian lungfish [8]. Exquisite illustrations of wax-model reconstructed embryos came with transparent overlay paper on which small ovals indicated the migration of “freien Mesodermzellen” (free mesoderm cells) from the neural tube and down the pharyngeal arches (Figure 1). It is only in hindsight, however, that we appreciate that Greil [8] was describing neural crest cells migrating to the major arteries and into the cardiac outflow tract [9]. That the neural crest cells play an important role in establishing separate channels for the pulmonary and systemic circulation has been described in a number of species thereafter [10,11]. To get to that insight, however, Greil’s descriptions were not considered. Instead, it was the use of quail-chick chimeras [12] that allowed Kirby and Stewart to understand that the neural tube (including premigratory neural crest) they had ablated contributes to the aorticopulmonary septum (which results from fusion of mesenchymal cushions) [13].

### 2.2. Cardio-Pharyngeal Mesoderm or Second Heart Field

Not only neural crest cells are migrating into the outflow tract. Early 20th century anatomists realized that the entire outflow tract in amniotic vertebrates is migrating towards the ventricle and Sir Arthur Keith found that “Dr. A. Greil, of Vienna, had published in the previous year (Morph. Jahrb., 1903, Bd. xxxi., p. 123) [14] a splendid research on the development of the heart of reptiles in which he demonstrated that the bulbus cordis, while a separate chamber at an early stage, becomes at a later stage overwhelmed by the musculature of the ventricles, and is thus not obliterated but incorporated as an intrinsic part of the ventricular system” [15] (the reference [14] is inserted by us). This incorporation was deduced from static anatomy of different developmental stages and it was by labelling the outflow tract of developing chicken that Maria de la Cruz added experimental support [16].

It is not only that cardiomyocytes change position within the heart. Virágh and Challice published in 1973 that the morphology of the cells at the arterial and venous poles of the embryonic mouse heart seemed similar to that of the cells of the neighboring mesoderm, from which they deduced that “splanchnic mesoderm” is added to the heart [17]. This notion has been thoroughly validated by lineage tracing experiments [18]. The second heart field, as this part of the splanchnic mesoderm is now called, also gives rise to facial musculature and thus establishes the link between craniofacial and cardiac malformations in syndromes such as DiGeorge syndrome [19]. The current tally of molecularly identified heart fields is three [20], and maybe even four if we accept the division of the second heart field into anterior and posterior parts [21,22]. A common issue has then emerged of whether the ‘splitting’ has become excessive and ‘lumping’ should be encouraged [23,24]. In this regard anatomy and molecular biology has surely seen many instances of parallel evolution.

### 2.3. Chamber Formation: Bulges, Segments, and Finally Balloons

**Figure 2:**
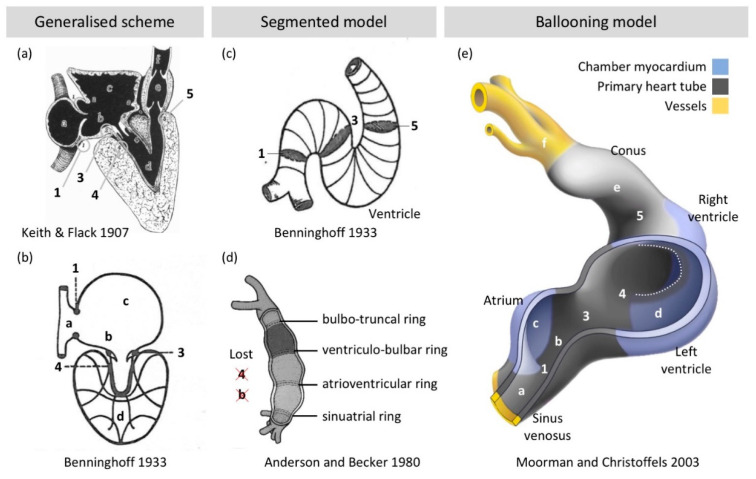
Building plan of the heart. (**a**) Keith and Flack [30] developed a “generalized” scheme of the vertebrate heart where the primitive parts (1,b,3,4,5) form a single domain (although their scheme is much inspired by the formed fish heart in which it is often the case that the atrioventricular part (3,4) is separated from the conal or bulbar valve area (5) by chamber myocardium). (**b**) The scheme of the cardiac conduction system by Benninghoff [31] is highly similar to that of (**a**) and while the labels a–d and 1,3,4 are inserted by us such that they correspond to (**a**), the primitive parts (1,b,3,4) still form a single domain. (**c**) Benninghoff [31], when conceptualizing the development of the conduction system from a comparative perspective (and maintaining that ectotherms do not have a conduction system comparable to that found in mammals and birds), emphasized the “Ostienringe” (1,3,5) as the precursors of future (“künftigen”) chamber junctions. Although this is still compatible with the ballooning model [29], the depiction of the junctions as entirely separate from each other (1,3,5) was perpetuated (**d**) to mean that everything between, for example, junctions 1 and 3 would be atrium, whereas Benninghoff’s model (**c**) does not exclude the presence of an “auricular canal” (label b in (**a**,**b**)), or remnant of the primary heart tube. (**d**) The segmented model presumes that parts b and 4 are lost and the precursors of all segments of the adult heart are present at the heart tube stage, which is therefore in conflict with the addition of cells from the second heart field, the “generalized” scheme (**a**) and the ballooning model (**e**) [29]. Image (**a**) is adapted from [30], (**b**,**c**) from [31], and (**d**) from [32].

Already Gaskell deduced that the myocardium between chambers was more embryonic-like in character [25,26]: “The study of the development of the heart shows that it is originally a simple tube with muscular walls from end to end of which waves of contraction pass; a portion of this tube expands to form the auricles and another portion to form the ventricle; these two being connected by an unexpanded part called the canalis auricularis”. This was corroborated by detailed anatomical studies of early embryos that described a primitive heart tube in which “the ventricles are outgrowths; or bulgings from the primitive cardiac tube” [27]. In the South American lungfish [28], for example, the atrium and ventricle are also “localized bulgings” and the atriums “expand markedly over the dorsal curvature of the heart, while the comparatively much slighter degree of ventricular expansion occurs wholly along the ventral curvature”. These descriptions strongly resemble our current notion of chamber development by “ballooning” of chambers from the outer curvature of the looped primitive heart tube [29]. Accordingly, the harmonizing views on vertebrate heart anatomy and development that were developed a century ago are still valid [30] (Figure 2).

It is then remarkable that the foundational descriptions were superseded by the notion that the primitive heart tube is made up of serially arranged primitive stages of the same segments that the adult heart has [33,34,35]. This ‘frame shift mutation’ in the understanding of the heart may have originated with Benninghoff [36], specifically in the schemes and simplifications he introduced for the earliest development of the conduction system [31]. Although his generalized scheme of the vertebrate heart (Figure 2b) is essentially the same as that of Keith and Flack [30] (Figure 2a), he presumed that junctions of the adult heart can be found back in the heart tube [31] (Figure 2c). Additionally, by illustrating the junctions as separate from each other (see the rings labelled 1, 3, 5 in Figure 2c), the single domain of primary heart tube myocardium, which is integral to the generalized model of the vertebrate heart, became segmented. This segmentation was taken very literal in later adaptations by Bob Anderson and others [33,34,37] in which the primary myocardium that makes up the entire heart tube was thought to be a tiny fraction of the tube only and organized in a series of rings (Figure 2d).

This segmented model (Figure 2d) is at odds with the anatomy of the heart tube (there is not a series of bulges), with the fact that a very substantial number of cells from the second heart field are added in later developmental stages (see Section 2.2) and that the heart tube comprises primary (non-chamber) myocardium only. It seems that ‘ventricle’ in the sense of a cavity was confused with the identity of the surrounding walls. Much of the ventricular (and atrial) cavity is indeed surrounded by ventricular (and atrial) wall, but a smaller part is surrounded by the primary heart tube (Figure 2e). In the case of Bob Anderson, it was the immunohistochemical detection of G1N2 and the remodeling of the atrioventricular junction it revealed [38] that caused him to discard the segmental model [39]. The ballooning model (or ‘bulging’ model in old parlance) is now extensively supported by data from human and model animal embryos [40,41,42].

## 3. Detailed Analyses of Ventricular Structure, But How Does It Relate to Function?

### 3.1. Myocyte Orientation of the Compact Wall

Pettigrew [43] documented in 1864 a surprisingly regular organization of the muscle of the left ventricle in various mammals, from a left-handed spiral in the subepicardial wall to a circumferential orientation in the mid-wall and a right-handed spiral in the sub-endocardial parts. In the mathematical analyses of Streeter [44], the spirals are geodesic lines, i.e., short routes around the cavity, and such a structure presumably would enable efficient pumping. Reptile hearts represent to some extent the ancestral condition of the mammalian heart [45,46] and Shaner [47] and Benninghoff [31,36] documented that reptile ventricles do not have the mammalian spiral organization of their compact wall. That allows for the conjecture that the spiraling configuration is related to some aspects of generating the greater cardiac output and higher systemic blood pressure that distinguishes mammals from reptiles.

From the observations of the spiral organization grew the notion that the left ventricular wall wrings like a towel in systole, which is supported by the tracking of fixed positions of the wall throughout the cardiac cycle. The ventricular wall is thick and for the ventricle to wring, layers closer to the cavity must move more than layers more to the outside. Consistently, sheets of aggregated muscle are found in much of the ventricular wall [48]. At least for the right ventricle, such sheets increase the risk of discontinuous electrical propagation, by which they can be a predisposing factor for arrhythmias [49]. Clonal analyses of ventricular cardiomyocytes of embryonic mice reveal that daughter cells are positioned much like pearls on a string [50,51]. Such configuration resembles geodesic lines. At early stages of development, the compact wall is only a few cells thick. Clonal analyses revealed a mostly circumferential orientation in the LV and a mostly longitudinal orientation in the RV. These analyses give much more detail than previous anatomical studies that suggested that the primitive fiber orientation is circumferential [52]. Recent X-ray-based tractography of fetal human hearts suggests the gradient of left-to-right handed orientation develop in the fetal period [53].

The spiral organization also spurred the notion that the entire ventricular mass is organized as a single band, as advocated by Torrent-Guasp, who famously cooked hearts and ‘unwound’ them [54,55]. The unwinding procedure starts with ripping the right ventricular free wall from the anterior interventricular sulcus and thus tearing naturally occurring bands of heart muscle. This attracted the ire of Bob Anderson who tried to impose nonsense-mediated decay of the concept [56]. Arguing against the single-banded ventricle may be one of the discourses he has spent the most words on. Evidence from developmental and comparative anatomy as well as functional studies has been amassed against the notion of a single-banded ventricle [57,58], but not everybody is convinced [59] and Bob Anderson will seemingly pick up the gauntlet every time [60].

Analyses of the spiraling organization is becoming quite sophisticated with the introduction of MRI and CT-based measurements enabling diffusion tensor imaging and tractography, respectively [53,61]. Nonetheless, one can reasonably posit that it is not obvious what the functional significance is of steeper or flatter helical angles, although experiments and case–control studies do show that hypertrophy of the ventricular walls associates with helical angles that deviate from normal [62,63]. The foundational analytic method that Streeter applied to the compact wall, did not apply well to the trabecular muscle on the luminal side of the ventricles. Later studies largely neglected the trabecular muscle. In clinical literature, however, the last three decades have seen a rapid increase in interest of the trabecular muscle as it has become associated with poor pump function [7,64,65,66,67,68].

### 3.2. Trabeculae, Compaction and Differential Growth as a Mechanism for Shape Change

The trabecular muscle of the left ventricle comprises only some 15% of the normal ventricular mass, the rest being compact wall [69]. While detailed analysis of the structure of the compact wall as geodesic lines can be made [61], the complex organization of the trabecular mass is more difficult to analyze [44]. Trabeculae are thought to increase wall stiffness [70] and negatively impact on the maximal stroke volume [71], in addition to facilitating thrombus formation like the trabecular muscle of the left atrial appendage [72] in atrial fibrillation [73]. The trabecular muscle component is proportionally greater than that of the compact wall in the hearts of embryos and in the cold-blooded vertebrates [74]. Subsequently, trabeculae have then been associated with immature [64,75] and primitive states [76,77]. A notion then formed of trabeculae being redundant at best and possibly even deleterious to pump function. In some individuals, the trabeculae are excessive and comprise more than 25% of the left ventricular mass [69]. These individuals can be diagnosed with left ventricular noncompaction [7,64,65,66,67,68]. The term noncompaction [64] relates to the hypothesis that the normal fetal ventricle is proportionally less trabeculated than the embryonic ventricle due to a putative process of compaction of the embryonic trabeculae into compact wall [78]. Noncompaction therefore assumes that compaction has failed [64]. It is therefore surprising that when thousands of individuals of the general population are surveyed, i.e., the study population has not been preselected on disease, a large fraction fulfills structural criteria for noncompaction while having normal pump function and no greater risk of severe outcomes [5,79,80,81,82,83]. Additionally, the evidence supporting the process of compaction taking place is surprisingly weak.

**Figure 3:**
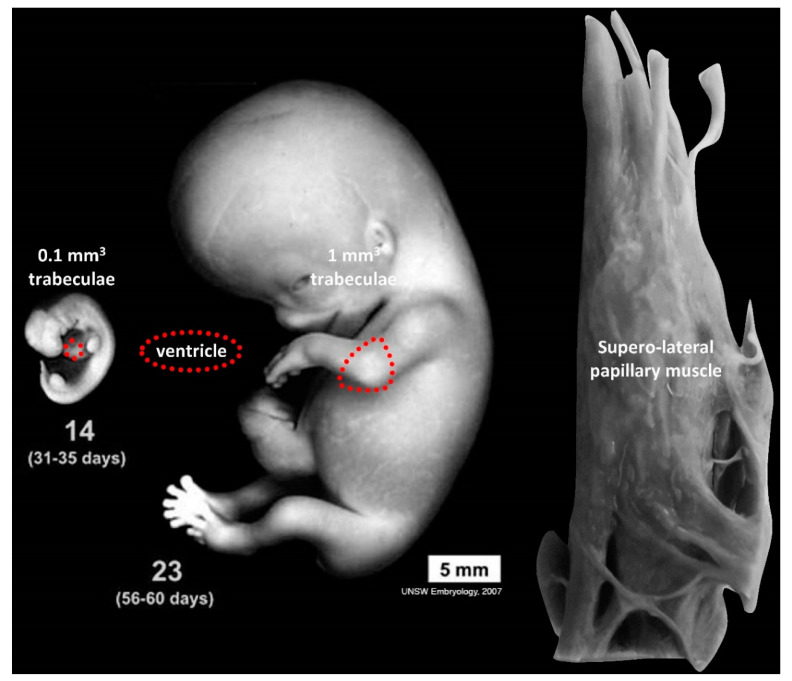
Growth of the cardiac ventricle (dashed red line) from a proportionally very trabeculated stage (14) to the end of embryonic development where compaction is presumed to have taken place (23) compared to the superior-lateral papillary muscle of the adult heart (which develops from embryonic trabeculae). From Carnegie stages 14 to 23, the volume of trabecular muscle increases approximately an order of magnitude [85] and then approximately five orders of magnitude to reach 10 g in the adult heart [69] (note that [85] included the ventricular septum in the volume of trabeculae and the numbers we show are a bit lower than in [85]). Images of embryos are adapted from Hill, M.A. (1 September 2020) Embryology Embryonic Development. Retrieved from https://embryology.med.unsw.edu.au/embryology/index.php/Embryonic_Development.

In the embryo, tissues and organs grow incredibly fast [84]. Volume measurements of trabeculae reveal an increase during development and not a decrease, as would be expected if compaction was an important process in the formation of the compact wall [85] (Figure 3). In the normal adult left ventricle, the trabecular component comprises approximately 5 g/m^2^ [69], which equals a greater volume than the entire embryo at the end of the embryonic period (8 weeks of gestation) [84]. A normal Carnegie stage 23 embryo and a normal papillary muscle, which derives from the embryonic trabeculae [86], are roughly similar in size (Figure 3). Furthermore, recent studies in mouse show that the growth of the compact wall is much more affected by diminished proliferation in the compact wall than diminished proliferation in the trabeculae [87]. That the trabeculae exhibit growth throughout gestation does not necessarily negate the existence of compaction (and thus noncompaction) in a narrow time window, but it does illustrate the tremendous scope for shape change by differential growth rates. Differential growth rates are evidently the driver of chamber formation and septation and, outside the heart, also the driver of morphogenesis of the limbs etc. [84,88]. Such “alternative splicing of information” has attracted the attention of Bob Anderson and while his erudition facilitated the notion of compaction [74,77], his quick pen now assigns only a minor role to compaction [89]. While the importance of compaction remains debated, the “mystery” of noncompaction [90] is being clarified because studies framed in the context of noncompaction have advanced our understanding of normal development of ventricular trabeculae [91,92,93,94] and their impact on health [5,79,80,95]. Thus, even if noncompaction turns out to be “the mitral valve prolapse of the 21st century” [68], i.e., excessively diagnosed, our knowledge of the biology of the trabeculae has increased in the process.

## 4. What Makes an Atrial Septum?

**Figure 4:**
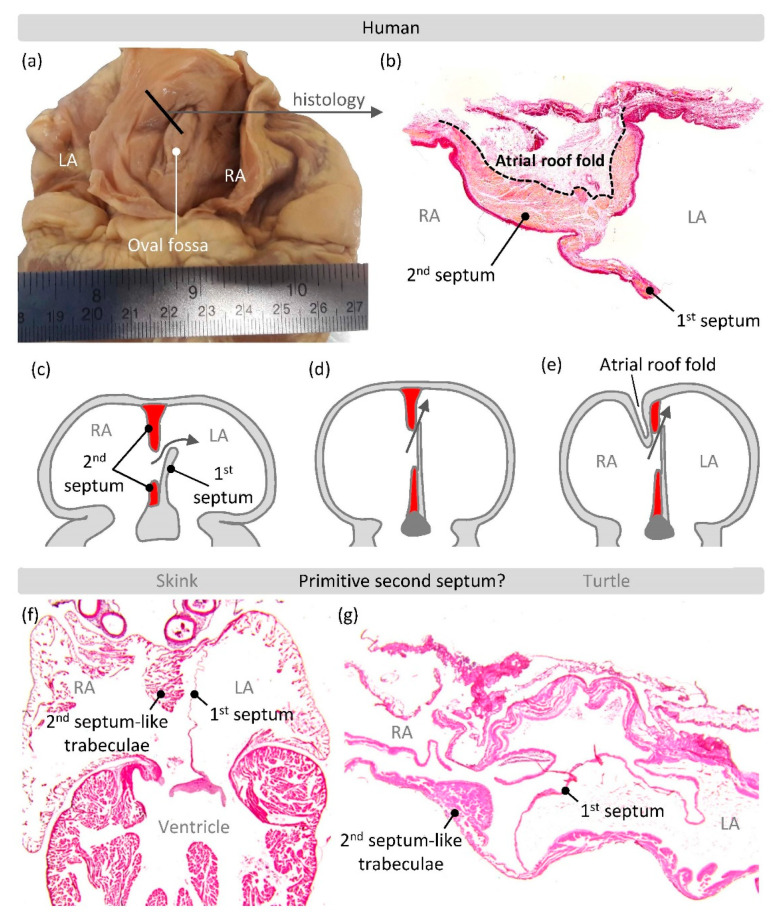
The atrial septum. (**a**) The atrial septum of the heart of an adult human exposed by a cut through the intercaval (sinus venarum) area. The thick black line indicates the part of the posterior-superior part of the septum that was sectioned with histology. (**b**) Histology showing the fold in the atrial roof (a very similar setting has been demonstrated in pig, for example [102,103]). (**c**,**d**) Schematic representations of the atrial septum of perinatal stages, with the secondary septum in red. Notice that the fold in the atrial roof is not cartooned. Adapted from [104,105]. (**e**) Example of schematic that makes a compromise between fold and septum, adapted from [106]. (**f**,**g**) Histology of reptiles showing a secondary septum-like aggregate of trabecular muscle in the right atrium of a skink ((**f**) horizontal section) and a turtle ((**g**) transverse section), adapted from [107].

Already in the late 19th century, Röse observed that the atrial septum of placental mammals was different from that of other vertebrates by having a circular depression, the oval fossa [96,97]. The floor of the oval fossa was shown to derive from the primary atrial septum and the rim of the oval fossa, then, became known as the secondary septum [98]. Ironically, the developmental studies of the time, including the one by Odgers [98], were cognizant that the largest part of the secondary septum, the posterior-superior part, was a folding-in of the atrial roof (Figure 4a,b). Therefore, this part was not a true septum as emphasized by Patten for example: “The walls of the right and left atria can then be separated from one another by merely dividing a little loose connective tissue, and the separation can be carried as far as practically to the margin of the limbus fossae ovalis. The whole of the dorsal and oral portion of the so-called septum secundum atriorum is therefore merely the result of the coaptation of adjacent portions of the walls of the two atria, and is not a true septum comparable to the septum primum” [99]. During the 20th century, the use of the term secondary septum persisted. In contrast, the original images were replaced with schematics that showed the fold as filled with heart muscle and the secondary septum now appeared like a septum [100,101] (Figure 4c,d).

Such anatomical “missense mutations” attracted the ire of Bob Anderson [108], who, after two decades of emphasizing fold over septum and knowing that the foundational descriptions were (largely) accurate, fumed that it “is difficult to explain why these facts did not make their way into standard accounts of cardiac development” [109]. Solace can be found in that recent accounts emphasize the folding in of the atrial roof [110] and even if they perpetuate the old erroneous view, the septum may become a fold in adaptations (see Figure 4c that appears to have inspired Figure 4d, which in turn got adapted such that the fold was included Figure 4e). It has been emphasized that in mouse there is more of a secondary septum (the fold is not very pronounced) [111] and many reptiles have a secondary septum-like structure suggesting that an actual septum rather than fold may be the evolutionary primitive condition [107] (Figure 4f,g).

Fold or septum, either is a barrier for blood flow and in this sense they are synonymous. The functional significance is that the dorsal rim is required for the closure of the foramen ovale after birth and the foramen ovale itself is likely an evolutionary adaptation to the long gestation that characterizes many placental mammals [112]. The evolutionary and developmental history of full atrial septation has recently been shown to have an additional component, namely a mesenchymal contribution called the vestibular spine or dorsal mesenchymal protrusion [112,113,114,115]. Accordingly, among the categories of atrial septal defects there is now a vestibular atrial defect and the prevalence of which seems underappreciated [116].

## 5. The Special Case of the Cardiac Conduction System

It is clearly so that more accurate knowledge is better, but at the same time it is certainly also true that the society of anatomical sciences has an epigenetic state predisposing it to spending many words on fine details. The cardiac conduction system, however, exemplifies a case where painstaking and tedious anatomical work led to insights of great clinical importance such as which parts of the heart can be operated on in corrective surgery [117,118,119] and which parts are most suitable for artificial pacing [120,121].

The cardiac conduction system comprises only a tiny fraction of the total myocardial mass of the heart and it took the advent of histology for Jan Purkinje to notice the large pale fibers with faint striations that we now call cardiac Purkinje fibers among the more readily identifiable chamber muscle [122]. Purkinje investigated the hearts of several species, but the fibers were most readily identifiable in sheep hearts [122]. Later, Wilhelm His junior described the bundle which penetrates the insulating plane and thus establishes communication between the atria and the ventricles [123]. Tawara expanded on this to show one system comprising the atrioventricular node, the His bundle, its bundle branches, and the Purkinje fibers it terminates in [124]. It is remarkable that the first descriptions of the conduction system with immunohistochemistry were firmly embedded in the foundational anatomical knowledge [125,126,127,128,129].

Regarding Tawara’s discoveries, it is interesting that Tawara was encouraged by his mentor Aschoff to investigate the atrioventricular bundle because of its perceived importance by “experimental physiologists” rather than anatomists [124]. Additionally, Tawara’s investigations into the connections between the bundle branches and the Purkinje fibers were essentially fruitless until Aschoff suggested he investigated sheep hearts (as Purkinje had) and “with this first sheep heart, I was able to completely solve the riddle” [124]. The efficacy of the sheep heart for making these discoveries, we now better understand, is that ungulates have extremely well-developed Purkinje systems [122,130]. That sheep are particularly suitable for the characterization of Purkinje fibers, exemplifies what is now called the Krogh principle, namely that for “a large number of problems, there will be some animal of choice or a few such animals on which it can be most conveniently studied” [131]. It is convenience, or relative ease of experimentation, that has drawn so much of molecular work to model systems such as fruit flies, zebrafish, and mice [132,133]. As more genomes are sequenced and molecular techniques become more broadly applicable [134], the number of species and biological problems that are investigated may again radiate out and many disciplines will then be needed, including anatomy. Hopefully, there will be future Bob Andersons who then preach the important anatomical insights to the uninitiated.
